# Oral Leukoplakia as It Relates to HPV Infection: A Review

**DOI:** 10.1155/2012/540561

**Published:** 2012-02-28

**Authors:** L. Feller, J. Lemmer

**Affiliations:** Department of Periodontology and Oral Medicine, University of Limpopo, Medunsa Campus, Medunsa, South Africa

## Abstract

Leukoplakia is the most common potentially malignant lesion of the oral cavity and can be categorised according to its clinical appearance as homogeneous or nonhomogenous. Tobacco and areca nut use, either alone or in combination are the most common risk factors for oral leukoplakia, but some oral leukoplakias are idiopathic. Some leukoplakias arise within fields of precancerized oral epithelium in which the keratinocytes may be at different stages of cytogenetic transformation. Leukoplakias may unpredictably regress, may remain stable, or may progress to carcinoma. There is a greater risk of carcinomatous transformation of idiopathic leukoplakia, of non-homogenous leukoplakia, of leukoplakia affecting the floor of the mouth; the ventrolateral surface of the tongue and the maxillary retromolar and adjoining soft palate (collectively called high-risk sites), of leukoplakia with high-grade epithelial dysplasia, and of leukoplakia in which the keratinocytes carry cytogenetic alterations associated with carcinomatous transformation. Although there appears to be some link between human papillomavirus (HPV) and oral leukoplakia, there is little evidence to support a causal relationship either between HPV infection and oral leukoplakia or between HPV-infected leukoplakic keratinocytes and their carcinomatous transformation.

## 1. Introduction

Leukoplakia is the most common potentially malignant lesion of the oral cavity [1, 2]. Leukoplakia is a term describing “a white lesion of the oral mucosa that cannot be characterized clinically or microscopically as any other defined oral disease entity” [3, 4]. At a World Health Organisation (WHO) workshop held in 2005, it was recommended that oral leukoplakia be defined as “a white plaque of questionable risk having excluded (other) known diseases or disorders that carry no increased risk for cancer” [5–7]. Oral leukoplakia needs to be distinguished from other predominantly white keratotic lesions including frictional keratosis and stomatitis nicotina, which do not have malignant potential [1, 2, 5–8].

About 70–90% of oral leukoplakias are related to smoking and areca nut use, either alone or in combination, and there is a direct relationship between the frequency and the duration of cigarette, pipe, or cigar smoking and the prevalence of oral leukoplakia [8, 9]. The factors implicated in the pathogenesis of idiopathic leukoplakia are unknown.

However it is possible that infection of the oral epithelium with human papillomavirus (HPV) and excessive consumption of alcoholic beverages may be associated with oral leukoplakia, but there is little evidence of a causal relationship between either HPV infection or alcohol, and oral leukoplakia [6].

It has been suggested that a definitive diagnosis of oral leukoplakia must be established by histopathological exclusion of other keratotic oral lesions that are recognised as specific entities, and by exclusion of any aetiological agents other than tobacco/areca nut use [7].

It is the opinion of the authors that these criteria are unrealistically limiting, ignoring as they do the possible roles of HPV, alcohol, chronic inflammation, and low-grade chronic frictional trauma in the pathogenesis of oral leukoplakia. Referring to the WHO definition of 2005 above, it is difficult to understand how such a definition could gain any useful currency since it is so “exclusive” that it leaves no rational guidance for the everyday diagnosis of oral leukoplakia.

## 2. Oral Leukoplakia: Clinical Aspects

According to its clinical appearance, oral leukoplakia can be categorised into two main clinical types: homogeneous and non-homogeneous. Either type may occur as an isolated lesion or as multiple lesions. The leukoplakic lesion can vary in size from a few millimetres to several centimeters [1, 2, 5–12].

Homogeneous leukoplakia is a uniformly white flat plaque with a smooth or relatively smooth surface; non-homogeneous leukoplakia may be nodular or verrucous having a wrinkled or corrugated surface or may be a mingling of white and red areas termed erythroleukoplakia [7, 10, 11].

The clinical appearance of oral leukoplakia may change over time. Some homogeneous lesions may become larger, or non-homogeneous, but most oral leukoplakias will remain stable or will regress, while some few will undergo carcinomatous transformation [13–15].

Oral erythroplakia, of all precancerous oral lesions, carries the greatest threat of malignant transformation. It has a velvety-red appearance, and about 50% of all cases of erythroplakia are already squamous cell carcinomata at the time of diagnosis. The erythroplakic component of oral erythroleukoplakia is identical to erythroplakia [16].

Proliferative verrucous leukoplakia, considered to be either a clinical subtype of non-homogeneous oral leukoplakia or to be a distinct clinical entity, is not strongly associated with smoking, is characterized by multiple leukoplakic lesions that affect wide areas of the oral epithelium, and may progress either to verrucous carcinoma or to squamous cell carcinoma [17–20]. In most cases, proliferative verrucous leukoplakia is recognised only late in its course since in its initial stages it is identical to an isolated leukoplakia [17–24].

## 3. Epidemiology of Oral Leukoplakia

The data from epidemiological studies on oral leukoplakia is inconsistent, most probably owing to differences in case selection criteria (house-to-house surveys, hospital surveys, age, gender, race, ethnicity, and tobacco use) and in methodology (diagnostic criteria, time of follow-up and whether or not the leukoplakia had previously been treated) [1, 10, 11].

Estimates of the global prevalence of oral leukoplakia range from 0.5% to 3.46%, and of the rates of carcinomatous transformation of oral leukoplakia from 0.7% to 2.9% [25]. Oral leukoplakia is more prevalent in India where persons smoke and practice the habit of tobacco and areca nut chewing more than elsewhere [26].

Oral leukoplakia is usually diagnosed in middle age, and its prevalence increases with age. About 10% of oral leukoplakias are idiopathic and the greater part of the remaining 90% is associated with the use of tobacco/areca nut [26]. Males are more often affected than females probably owing to the greater prevalence of tobacco use by males [8]. The buccal mucosa is affected in 25% of cases, the mandibular gingiva in 20%, the tongue in 10%, the floor of the mouth in 10%, and other oral sites account for the remainder [13].

The literature on the relationship between race and oral leukoplakia is sparse. In a South African study of archived histopathological material, 86% of oral leukoplakias were from whites, 9% from blacks, and 5% from Asians, despite the fact that the vast majority of South Africans are black [27]. This is not easy to explain. Bearing in mind that the study was on histopathological material, an explanation may be that blacks tend to postpone seeking medical treatment until the leukoplakia has already undergone carcinomatous transformation, thus skewing the statistics away from the leukoplakia [27], or because black people in South Africa may smoke less than white people.

## 4. Epithelial Dysplasia and Oral Leukoplakia

The reported prevalence of epithelial dysplasia in oral leukoplakia ranges from 5% to 25% [8]. Dysplasia is more frequent in non-homogeneous than in homogeneous leukoplakia [11], and it is probable that dysplasia is the histopathological expression of genomic and molecular alterations in a field of keratinocytes [28, 29].

The presence of epithelial dysplasia is a marker of the malignant potential of oral leukoplakia, and the risk of an individual leukoplakic lesion to progress to carcinoma increases with the increase of the grade of the epithelial dysplasia [11, 12, 14, 30].

However, some dysplastic oral leukoplakias can remain stable or even regress, while some oral leukoplakias without epithelial dysplasia will indeed progress to carcinoma [5, 11, 12]. In one study 36% of dysplastic oral leukoplakias progressed to squamous cell carcinoma, but a substantial proportion of 16% of oral leukoplakias without epithelial dysplasia at the time of initial biopsy also progressed to carcinoma [31]. The risk of progression to carcinoma of leukoplakias with moderate and severe dysplasia is estimated to be twice as great as for oral leukoplakias with simple epithelial hyperplasia or with mild dysplasia [14].

Treatment of dysplastic oral leukoplakia by excision, by laser or by cryosurgery, or by topical or systemic chemotherapy does not eliminate either the risk of relapse or recurrence, or the risk of carcinomatous transformation [1, 5, 6]. The estimated recurrence rate of oral leukoplakia may be as high as 30% [32], and squamous cell carcinoma develops at 12% of sites of treated leukoplakia [15]. In a study investigating the pattern of carcinomatous transformation of oral leukoplakia and oral erythroplakia, 36% of carcinomata developed at the same site, 49% at contiguous sites, and 15% at oral sites remote from the preexisting lesions [33].

It is evident from this data that some cases of treated oral leukoplakia are unpredictably destined to recur or to undergo carcinomatous transformation, and that there are not yet any diagnostic methods available (clinical, histological or molecular), to confidently identify these cases [1].

Epithelial dysplasia in oral leukoplakia is a useful marker of the risk of carcinomatous transformation and is an important guide to clinical management [1, 11, 28]. However, since dysplasia can remain stable for long periods, it cannot be used with confidence as a predictor of carcinomatous transformation [14, 29]. Moreover, as the histological exercise of grading of epithelial dysplasia is highly subjective with low interpersonal and intrapersonal reproducibility [16, 34, 35], and as an incisional biopsy cannot be representative of an entire lesion [34, 36], a histopathological report of any degree of epithelial dysplasia or of the absence of epithelial dysplasia must be viewed with caution.

## 5. The Natural Course and the Malignant Potential of Oral Leukoplakia

The progression of oral leukoplakia to carcinoma is unpredictable but is relatively infrequent with an estimated overall risk of less than 2% per year [5, 6, 13, 26]; if progression occurs, it may take a few months or many years [8]. The carcinomatous transformation of oral leukoplakia is not predictably associated with tobacco smoking [33], and the frequency of carcinomatous transformation of idiopathic leukoplakia is higher than that of tobacco-associated leukoplakia [11, 26].

In populations where smoking, the use of smokeless tobacco, reverse smoking, and the use of areca nut are very prevalent, most squamous cell carcinomata arise from preexisting leukoplakias; while in populations with a lower prevalence of these habits, most squamous cell carcinomata arise *de novo *in normal-looking epithelium [26]. It has been suggested that squamous cell carcinoma arising *de novo* usually runs a more aggressive course and has a less favourable prognosis than squamous cell carcinoma arising from preexisting leukoplakia [12, 26], but a recent study has demonstrated little difference [37]. Sometimes squamous cell carcinoma arises *de novo *in close proximity to oral leukoplakias [8].

Non-homogeneous leukoplakia has a greater risk of carcinomatous transformation (20–25%) than homogeneous leukoplakia (0.6–5%) [11, 13]. Most leukoplakias either remain stable or will regress [13, 15]. However, if proliferative verrucous leukoplakia is considered as a distinct entity, most such cases progress to carcinoma [18, 24].

The rates of progression of large oral leukoplakias (>5 mm) and of leukoplakias at sites in the mouth known to be at most risk of developing carcinoma (floor of mouth, ventrolateral surface of tongue, and maxillary retromolar/soft palate region) are greater than for smaller leukoplakias or for leukoplakias at other sites in the mouth [1, 11, 13, 33, 38]. The increased risk of carcinomatous transformation of oral leukoplakia at high-risk sites is not entirely a function of the degree of dysplasia. It is also dependent upon as yet undefined characteristics of the location of the leukoplakia since the rate of carcinomatous transformation of dysplastic leukoplakias at high-risk sites is greater than the rate of transformation of equally dysplastic leukoplakias at other sites [38].

There is evidence that clearly suggests that some leukoplakias arise from cytogenetically altered transformed keratinocytes within fields of precancerized oral epithelium. Keratinocytes of oral leukoplakia show cytogenetic changes including alterations in the p53 tumour suppressor gene, aberrations in their DNA content, and loss of heterozygosity (LoH) at chromosomal regions of candidate tumour suppressor genes [20, 33, 39–45]. LoH at either 3p or at 9p occurs frequently in keratinocytes of oral leukoplakia and is associated with carcinomatous transformation of these lesions [33, 41, 42, 44, 45]. Additional cytogenetic alterations to the keratinocytes in the precancerized field referred to above may result in the evolution of one or several keratinocytes containing a complete set of cytogenetic alterations of a cancerous phenotype, and in the subsequent development of squamous cell carcinoma [41, 46, 47].

However, some precancerous oral leukoplakias in which cytogenetic alterations in the keratinocytes cannot be demonstrated, nevertheless undergo carcinomatous transformation [41, 42]. The pathogenic mechanisms that bring about the progressive transformation of these keratinocytes to carcinomatous cells are yet to be elucidated. Most oral leukoplakias are benign in nature and will remain stable or will regress [28, 32]. These leukoplakias probably have a different aetiopathogenesis to precancerous leukoplakias and probably do not have the cytogenetic characteristics of precancerous leukoplakias. What is certain, however, is that leukoplakias with malignant potential and those without malignant potential cannot be distinguished clinically [1].

## 6. Human Papillomavirus and Oral Leukoplakia

Human papilloma viruses (HPVs) are strictly epitheliotropic and infect either cutaneous or mucosal squamous epithelium, depending upon their genotype [48, 49]. Those that infect mucosal epithelium have been categorized into high-risk types (e.g., HPV-16, 18, 31, 33, and 35) based on their epidemiological association with carcinoma of the cervix uteri, or into low-risk types (HPV-6, 11, 13, and 32) [50]. These categories have been universally adopted for use in studies of the oncogenic significance of HPV infection at all anatomical regions of the upper aerodigestive tract.

Low-risk HPV genotypes have been implicated in the pathogenesis of the benign oral proliferative epithelial lesions, squamous cell papilloma, common wart (verrucous vulgaris), condyloma acuminatum, and focal epithelial hyperplasia (Heck disease); while high-risk types have been associated with precancerous and cancerous oral and oropharyngeal epithelial lesions [49, 51–56].

There is an extreme variation in the reported prevalence of HPV infection in oral precancerous and cancerous lesions ranging from 0% to 100% [57, 58]. This is owing to differences in sampling and HPV detection methods, to differences in ethnicity, geographic locations, and sample size of the subjects examined, and to the inappropriate grouping together of different lesions from different anatomical locations of the mucosa of the upper aerodigestive tract [49, 51, 58–64].

Many studies investigating the association of HPV and squamous cell carcinoma of the mucosa of the upper aerodigestive tract used PCR techniques for detection of HPV DNA without also quantifying the DNA viral load. PCR can detect extremely small fragments of DNA that may represent either contamination of the sample or biologically insignificant HPV infection [51, 57, 58]. These findings have been reported as if they were pathogenically significant.

Whether these are legitimate findings or are the results of inconsistencies and errors in methodology, several HPV genotypes have been detected in precancerous oral lesions. High-risk HPV genotypes, in particular HPV-16, have been reported to be the most prevalent in oral leukoplakias, including proliferative verrucous leukoplakia [55, 65]. Other reports implicated low-risk rather than high-risk HPV genotypes in oral leukoplakia [54, 63], and yet others assert that oral leukoplakia is coinfected with a variety of HPV genotypes [49, 55, 66]. In a meta-analysis of data from 94 studies of a total of 4580 specimens, Miller and Johnstone [63] determined that the likelihood of HPV being detected in precancerous oral lesions is 2 to 3 times greater and in oral squamous cell carcinoma is 4 to 5 times greater than in normal oral mucosa. The prevalence of HPV in normal oral mucosa, in nondysplastic leukoplakias, in dysplastic leukoplakias and in other precancerous intraepithelial oral neoplasms, and in oral squamous cell carcinoma is likely to be 10%, 20.2%, 26.2%, and 46.5%, respectively [63].

This suggests that there may be some link between HPV infection and oral precancerous and cancerous lesions. As E6 and E7 oncoproteins of high-risk HPV genotypes have the capacity to mediate carcinomatous transformation of infected keratinocytes by inactivating cellular p53 and Rb tumour suppressor pathways [50, 52], HPV may play either an oncogenic or a co-oncogenic role in some HPV-infected precancerous and cancerous epithelial neoplasms.

In fact, HPV-16 has been found to be causally associated primarily with squamous cell carcinoma of the palatal tonsils [67–70] in a subset of subjects who are younger, consume less tobacco, are more engaged in high-risk sexual behaviour (great number of lifetime sexual partners and practicing oral-genital sex), have higher HPV-16 serum antibody titers, and have a better disease-free survival and overall survival rates than subjects with HPV-cytonegative oropharyngeal squamous cell carcinoma [51, 64, 67–70]. The cells of HPV-cytopositive oropharyngeal carcinoma in these subjects have a distinct molecular profile [50]. The cells of squamous cell carcinoma causally associated with HPV express E6/E7 oncoproteins. They frequently demonstrate viral integration within the cellular genome with the presence of intact E6 gene. They exhibit high viral load, reduced expression of Rb proteins, functional overexpression of p16 INK4A, unmutated p53 gene, and loss of heterozygosity (LoH) at chromosomal loci 3p, 9p and 17p is infrequent [51, 68–74]. In contrast, HPV-cytonegative oropharyngeal squamous cell carcinoma is characterized by p53 gene mutations, by frequent LoH at 3p, 9p, and 17p, by decreased levels of p16INK4A and by normal or increased levels of Rb proteins [50, 51, 71].

Recent meta-analyses and comprehensive studies [60, 62, 67, 75] show little or no causal association between HPV and oral squamous cell carcinoma in contrast to the strong association between HPV and oropharyngeal squamous cell carcinoma. HPV-16 cytopositive oral squamous cell carcinoma is characterized by a low viral load, by infrequent viral integration, and the cancerous cells seldom contain active transcriptional E6/E7 mRNA [72, 76]. However, it is possible that, in HPV-cytopositive oral squamous cell carcinomata that do not express E6/E7 mRNA, E6/E7 oncoproteins may well have participated or have had a complementary role in the initial transformation, but then phased out [77].

With oropharyngeal carcinoma as the model, a causal association between HPV and cancerization in oral epithelium is likely if the cells of the lesion contain HPV DNA expressing E6 and/or E7 mRNA [71], if there is viral integration within the cellular genome [74], and if there is a high viral load (>1 copies per cell). A limited biological significance of the virus in the process of transformation can be deduced if there is a low-copy number (<1 copy per cell), or if there is no transcriptional activity of E6 and/or E7 mRNA [60, 71]. Nevertheless, although integration of HPV DNA into the cellular genome is a strong indication of the oncogenic potential of the virus, transcription of HPV-16 E6/E7 mRNA has been shown to occur in oropharyngeal carcinoma without integration of viral DNA, the virus being in an episomal form [69].

It is well established that non-homogeneous leukoplakias more frequently undergo malignant transformation than homogeneous leukoplakias, yet it appears that HPV is found more commonly in homogeneous than in non-homogeneous leukoplakia [54, 78]. Nevertheless the role of HPV in the pathogenesis of oral leukoplakia and in its progression to carcinoma is unclear since there is a low viral load in HPV-cytopositive precancerous and cancerous oral lesions, and viral integration is seldom found [72, 76]. It is possible that HPV DNA in oral leukoplakia and in oral squamous cell carcinoma may be oncogenically insignificant. Alternatively the HPV may have superinfected keratinocytes already initially transformed and may thus additively or synergistically promote later stages of transformation [49, 51, 69].

Little is known about the E6 and E7 proteins of low-risk HPV either with regard to their role in the pathogenesis of HPV-infected oral leukoplakias, or with regard to their role in the carcinomatous transformation of some leukoplakias. It is possible that as in other HPV-associated benign proliferative oral epithelial lesions, E6 and E7 proteins of low-risk HPV types found in oral leukoplakia may stimulate suprabasal postmitotic infected keratinocytes to reenter the S-phase of the cell cycle resulting in epithelial proliferation and disturbed maturation, without causing the genomic instability possibly associated with subsequent cell transformation. This mechanism may be a co-determinant of the development of the leukoplakia, but there is no concrete evidence to support this.

## 7. Treatment

As oral leukoplakia is potentially malignant, and as some leukoplakias will unpredictably progress to carcinoma, ideally all oral leukoplakias should be treated. When dealing with two or three accessible circumscribed lesions, the treatment of choice is surgical excision. For multiple or for large leukoplakias where surgical treatment would be impractical because it would result in unacceptable deformities or in functional disabilities, treatment can be by cryosurgery, laser surgery, or by the use of topical bleomycin. However, regardless of the extent of the lesion or of the modality of treatment, in as many as 30% of treated cases, leukoplakias will recur and treatment will not prevent the progression of some leukoplakias to squamous cell carcinoma [5, 15, 21, 32].

Idiopathic leukoplakia, non-homogeneous leukoplakia, leukoplakia affecting high-risk oral sites, and leukoplakia showing moderate or severe grades of epithelial dysplasia and particularly leukoplakias, in which a combination of these factors affect the risk of carcinomatous transformation, should be treated aggressively. Any changes in colour, texture or size, and appearance of additional leukoplakias at new oral sites are advance warning of the possibility of carcinomatous transformation.

## 8. Summary

Leukoplakia is the most common potentially malignant lesion of the mouth. It may unpredictably regress, may remain stable, or may undergo carcinomatous transformation. Those leukoplakias that are committed to a cancerous pathway most probably arise within a field of precancerized epithelium consisting of keratinocytes at different stages of cytogenetic transformation. This may explain the high rate of recurrence of oral leukoplakia despite treatment, and why some leukoplakias progress to carcinoma.

Many studies have reported the presence of HPV DNA in oral leukoplakias. However, there is not enough evidence to prove any casual association either between HPV and the development of oral leukoplakia, or between HPV and the progression of oral leukoplakia to carcinoma. The nature of the link between HPV infection and oral leukoplakia is as yet unknown.
